# Markov blankets in the brain

**DOI:** 10.1016/j.neubiorev.2021.02.003

**Published:** 2021-06

**Authors:** Inês Hipólito, Maxwell J.D. Ramstead, Laura Convertino, Anjali Bhat, Karl Friston, Thomas Parr

**Affiliations:** a Humboldt-Universität zu Berlin, Department of Philosophy & Berlin School of Mind and Brain, Germany; b Wellcome Centre for Human Neuroimaging, University College London, United Kingdom; c Division of Social and Transcultural Psychiatry, Department of Psychiatry, McGill University, Montreal, Quebec, Canada; d Culture, Mind, and Brain Program, McGill University, Montreal, Quebec, Canada; e Institute of Cognitive Neuroscience (ICN), University College London, London, United Kingdom

**Keywords:** Markov blankets, Dynamic causal modelling, Boundaries, Canonical microcircuit

## Abstract

Recent characterisations of self-organising systems depend upon the presence of a ‘Markov blanket’: a statistical boundary that mediates the interactions between the inside and outside of a system. We leverage this idea to provide an analysis of partitions in neuronal systems. This is applicable to brain architectures at multiple scales, enabling partitions into single neurons, brain regions, and brain-wide networks. This treatment is based upon the canonical micro-circuitry used in empirical studies of effective connectivity, so as to speak directly to practical applications. The notion of effective connectivity depends upon the dynamic coupling between functional units, whose form recapitulates that of a Markov blanket at each level of analysis. The nuance afforded by partitioning neural systems in this way highlights certain limitations of ‘modular’ perspectives of brain function that only consider a single level of description.

## Highlights

• We leverage the idea of ‘Markov blanket’ as a statistical boundary to provide an analysis of partitions in neuronal systems.
• We show this partition is applicable to multiple scales, from single neurons, brain regions, and brain-wide networks.
• Based on the canonical micro-circuitry, our treatment has practical applications for effective connectivity.
• Our proposed partition highlights the limitations of ‘modular’ proposals considering a single level of description.

## Introduction

Scientific investigation in neurobiology often begins – perhaps only implicitly – by partitioning the brain into functional units. This is important, as it is only by segregating parts of the brain from other parts that we can start to ask questions about how they interact. While the most obvious partition of neural systems is into individual neurons, the same approach can be applied over a range of spatiotemporal scales.

The division of the cortical surface into Brodmann areas represents one such carving up of neural tissue (Brodmann, 2007; (Zilles and Amunts, 2010). Brodmann maps have enduring practical implications. For example, the Talairach Atlas (Talairach and Szikla, 1980a, 1980b), commonly in use in neuroimaging, may be seen as a direct descendent. In this setting, the assumption is that brain function depends upon interactions between architectonically defined brain regions (Lazar, 2008). This assumption underwrites the study of connectivity in the brain, as we need to know what is connected to what. Effective connectivity studies go as far as to distinguish between connections that are ‘intrinsic’ or ‘extrinsic’ to a given region (or cortical column) (Tsvetanov, Henson et al. 2016; Zhou et al., 2018). Again, this rests upon drawing boundaries around parts of a brain. Our focus in this paper is on how such boundaries are licensed.

A prominent justification for drawing boundaries – from the last century – is the ‘modularity of mind’ paradigm (Fodor, 1983), which itself inherits from the phrenology of the preceding century (Gall and Lewis, 1835). This conceptualisation of cognitive processes depends upon discrete cognitive units that interact with one-another, which might manifest in the tissue engaged in cognitive operations. Broadly speaking, modularity in the brain refers to some form of segregation of neuronal processing in specialised modules conducting computation in isolation from the rest of the system (Coltheart, 2011). However, more recent perspectives, based upon stochastic non-equilibrium systems, offer a simpler perspective in terms of factorisation (Parr et al., 2020a, 2020b). Specifically, conditional independency between two parts of a system lends it a modular appearance. An important limitation of the modular paradigm is that it typically only considers a single level of description, neglecting the rich intrinsic and extrinsic dynamics across regions and microcircuits. In addition, the philosophical assumptions of modular perspectives on neuronal organisation have been criticised (Friston, 2002; Colombo, 2013; Palecek, 2017; George and Sunny, 2019; Hipólito and Kirchhoff, 2019). In short, this calls for a more nuanced treatment of partitions and functional interactions. In this paper, our focus is upon the conditional dependency structure in neural systems.

A growing literature leverages the *Markov blanket* construct (Pearl, 1998) to formalise dynamic coupling in physical and biological systems (Friston, 2019a, 2019b, Hipólito, 2019; Ramstead et al., 2018, 2019; Palacios et al., 2020; Kirchhoff et al., 2018). This construct is a description of the dependencies within and between random dynamical systems – like the brain – that sets a boundary between the inside and outside of each system. Here, we focus upon the Markov blankets implicit in models used practically in characterising brain function. Specifically, we examine the dynamics implied by neural mass models^1^ of the kind that underwrite Dynamic Causal Modelling (DCM) (Bastos, Usrey et al. 2012; Moran, Pinotsis et al. 2013). Building from this to the connectivity of a canonical cortical microcircuit, we set out a series of Markov blanketed structures at increasing spatial scales.

This approach endorses the segregation of the brain into regions but also emphasises the absence of a privileged scale of description at which 'modules' might be defined. By selecting a Markov blanket, we implicitly identify the variables that define the simplest element of our system at a given scale. It follows that, depending on the scale of interest, the variables comprising the Markov blanket will be different. For a single neuron, the blanket includes the presynaptic and postsynaptic membrane potentials that mediate its interactions with other neurons. For cortical columns, the blanket will include neural populations mediating interactions between different columns. In principle, the identification of functional boundaries can proceed at finer (ion channels and molecules) and coarser (networks, brains, and people) scales.

Our primary focus here is upon the specific interpretation of hierarchy as a spatial progression. This lets us zoom in and out in at different levels of neuronal architecture—emphasising its status as a multiscale system. It is the relationship between these spatial scales that is lacking in modular accounts, which tend to focus upon relationships between entities defined at a specific scale. Specifically, we consider hierarchical laminar connectivity (Fig. 5) and hierarchy as a progression of scales. Although our proposal is consistent with the principle of progression of scales, we do not commit to the assumption of Hierarchical Modules in the Network (HMN), i.e., a fractal hierarchy of neuronal systems and the global integration of functionally segregated units (Sporns, 2006; Clauset et al., 2008). Meunier et al., 2010). We suppose that the hierarchical mechanistic mind (HMM) formulation is conceptually better suited to study of the embodied, situated human brain (Badcock et al., 2019a, 2019b). In the HMM, the brain is described as a complex adaptive system that functions to minimize the entropy of our sensory and physical states via action-perception cycles that depend upon (spatially and temporally) hierarchical neural dynamics. We follow Hilgetag and Goulas (2000) in seeking a construct that is more specific than ‘hierarchy’ – for a more precise understanding of the organizational principles of functional anatomy.

While identifying blankets at each level may seem an abstract exercise, it has important implications for empirical neuroscience. Specifically, it offers an important part of the conceptual analysis we need to ensure our hypotheses make sense (Nachev and Hacker, 2014). For example, if we want to know whether condition specific differences in measured brain activity are mediated by changes in ‘intrinsic’ or ‘extrinsic’ connectivity (Zhou et al., 2018), we need to be able to define what we mean by these terms, and to say what they are intrinsic or extrinsic to. We aim to make this explicit in a series of examples.

The aim of this paper is to argue that an appeal to the Markov blanket construct provides a formal basis for partitioning the brain into functional units – from individual neurons to functional assemblies of neurons, through to independent brain regions and networks of regions. In particular, we will argue that a recursively iterated version of the formalism, where each component of a Markov blanketed system is itself a Markov blanketed system, is apt for the task. This paper comprises four parts. The first provides a brief overview of the Markov blanket construct and its relevance to a dynamical setting. The second section zooms in on the individual neurons and illustrates how synaptic dynamics conform to the conditional independence structure of a Markov blanket. The third takes a more detailed look at the asymmetries of the neuronal Markov blanket, and emphasises the need for these to be replicated at the network level. The fourth section shows how the same structure is recapitulated at larger spatial scales.

## 1 Markov blankets

**Fig. 1:**
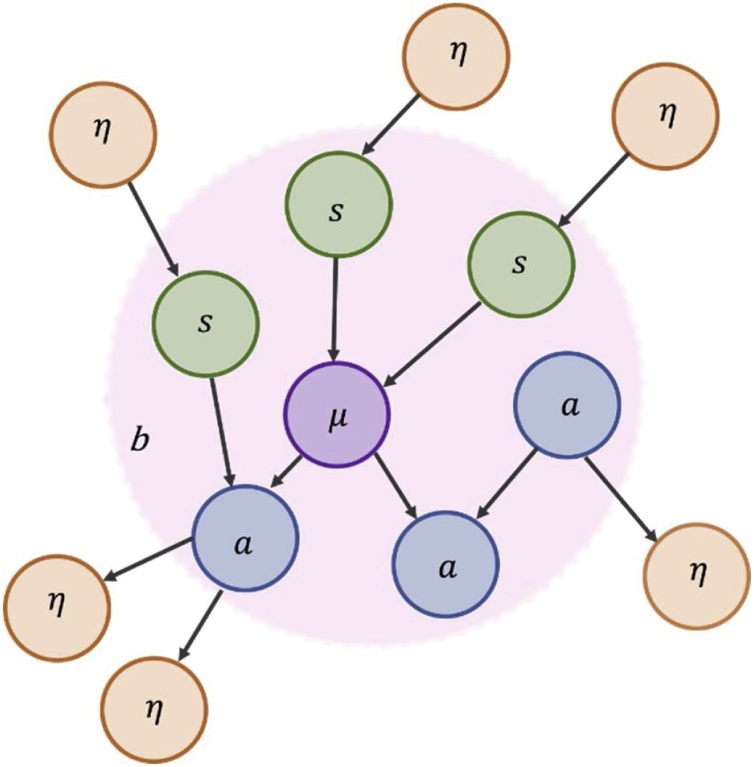
Markov blanket. A Markov blanket highlights open systems exchanging matter, energy or information with their surroundings. Variables η are conditionally independent of variables μ by virtue of its Markov blanket (*b*). If there is no route between two variables, and they share parents, they are conditionally independent. Arrows go from parents to children. We will use the colour-scheme in this figure consistently through subsequent figures.

The Markov blanket construct, which underwrites the current proposal, was introduced into the literature by Pearl (1998) in the context of statistical inference. To distinguish a set of systemic (or internal) states from their embedding environment (of external states), a third set of states are implied^2^ . These are blanket states (Friston, 2013). The Markov blanket consists of sensory states, which affect but are not affected by internal states; and active states, which affect but are not affected by external states (Fig. 1)^3^ . This implements conditional independence between internal and external states, under mild assumptions.

By Markov blankets, we mean a partition that complies with the conditional dependency structure of Eq. (1) and the dynamics of Eq. (2). It is important to keep in mind that a Markov blanket is not necessarily a physical boundary such as a cell membrane, but rather a statistical one defined by variables that are conditionally independent of each other. There is an ongoing debate about whether these constructs should be interpreted in a realistic way, as a literal description of the brain, or in an instrumentalist way, as a useful tool to gain insight over the neuronal and cognitive activity, without assuming the existence of Markov blankets in the brain. Our discussion is orthogonal to this issue; but see (Andrews, 2020; Bruineberg, Dolega et al. 2020; Ramstead et al., 2020; van Es and Hipólito, 2020) for discussion.

A Markov blanket (*b*) around internal states *μ* – where all other (external) variables are labelled *η* – is defined as the set of variables that renders *μ* conditionally independent from *η*. Mathematically, this is written as follows:

(1) μ⊥η|b⇔p(μ,η|b)=p(μ|b)p(η|b)

Eq. (1) illustrates this dependency structure in the factorisation of the joint distribution conditioned on blanket states into two conditionally independent distributions; by definition, two variables are conditionally independent if and only if their joint probability, conditioned on some third variable, is equal to the product of their marginal probability conditioned on that third variable. It is common to speak of the random variables separated in this way by Markov blankets – and the associated conditional dependencies – in terms of ‘parents’ and their ‘children’, where ‘parent’ nodes cause their children. A Markov blanket is then the set of the parents, the children, and the parents of the children of the variable in question. An alternative way to frame this is to think of the parents as mediating the influence of external states on internal states (i.e., sensory states) and the children (and their parents) as mediating the influence of internal states on external states (i.e., active states). This suggests a separation of blanket states into active (*a*) and sensory (*s*) states.

**Fig. 2:**
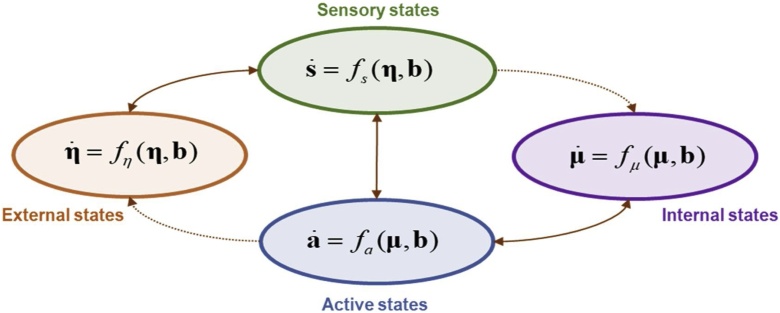
This schematic illustrates the partition of states into internal states (purple) and hidden or external states (orange) that are separated by a Markov blanket – comprising sensory (green) and active states (blue). Specifically, it focuses on the dynamical formulation of Eq. (2). Directed influences are highlighted with dotted connectors. Autonomous states are those states that are not influenced by external states, while particular states constitute a particle; namely, autonomous and sensory states – or blanket and internal states. Sensory states, active states and internal states comprise the particular states that are constitutive of a functional neuronal unit (for more detail see Hipólito, 2019).

In a dynamical setting^4^ (Friston et al., 2020b), Eq. (1) means that the average (represented in bold) rate of change of each component of a Markov blanketed system can only depend on two other sorts of state in order to preserve the structure of Eq. (1). This is shown in Eq. (2) and Fig. 2:

(2) $$\begin{array}{c} \dot{\text{μ}}=f_{\mu }\left(\text{μ,s,a}\right)\\ \dot{\text{a}}=f_{a}\left(\text{μ,s,a}\right)\\ \dot{\text{η}}=f_{\eta }\left(\text{η,s,a}\right)\\ \dot{\text{s}}=f_{s}\left(\text{η,s,a}\right) \end{array}$$

Eq. (2) means that the flow of internal and external states does not depend upon one another; i.e., that internal states cannot influence sensory states, and that external states cannot influence active states. Additionally, note that the Markov blanket structure is preserved if dependencies are lost (e.g., if the active states were not influenced by sensory states), but not if they are gained, since that would – in most circumstances – destroy the conditional independence. We will see over the next few sections that this structure can be identified at numerous levels neuronal organisation; especially in dynamical formulations of neuronal circuits, based upon neural mass models (David and Friston, 2003; Pinotsis et al., 2014; Moran et al., 2013).

Before proceeding, it is worth briefly unpacking the reason for the names of the variables. While the Markov blanket formulation in general applies to any random variables, recent work has leveraged Markov blankets to talk about the structure of exchanges between an organism and its environment (Friston, 2013; Kirchhoff et al., 2018; Parr and Friston, 2018a, 2018b) and to describe self-organisation across spatial and temporal scales (Hipólito, 2019; Ramstead et al., 2018; Palacios et al., 2017). In this context, we associate the variable of interest with the internal states of a Markov blanket, which allows us to think of the ‘parents’ of that variable as mediating the influence of external states on internal states (i.e., as sensory states) and of its ‘children’ and the ‘parents of the children’ as mediating the influence of internal states on external states (i.e., as active states). This conception of the Markov blanket as the mediating influence of external states on internal states through the effects of sensory and active states resonates with the action-perception cycles typically considered in cognitive systems (Fuster, 1990; Parr and Friston, 2017, 2018a, 2018b). This is the reason for the words ‘active’ and ‘sensory’. While it may seem strange to use these terms for interactions at cellular or network levels, it should be emphasised that these are simply names for statistical constructs.

## 2 Neurons and their Markov blankets

In this section, we consider the partition of brain tissue into neurons. From a dynamical perspective, this means finding equations of motion consistent with Eq. (2) and Fig. 2. We know that synaptic dynamics conform to the dependency structure of a Markov blanket, as the internal states (e.g., conductance of ion channels) of one neuron are distinguishable from the same states of other neurons but interact through presynaptic and postsynaptic voltages. The implied partitioning of tissue into Markov blanketed neurons allows neurons to change their behaviour without losing their identity.

**Fig. 3:**
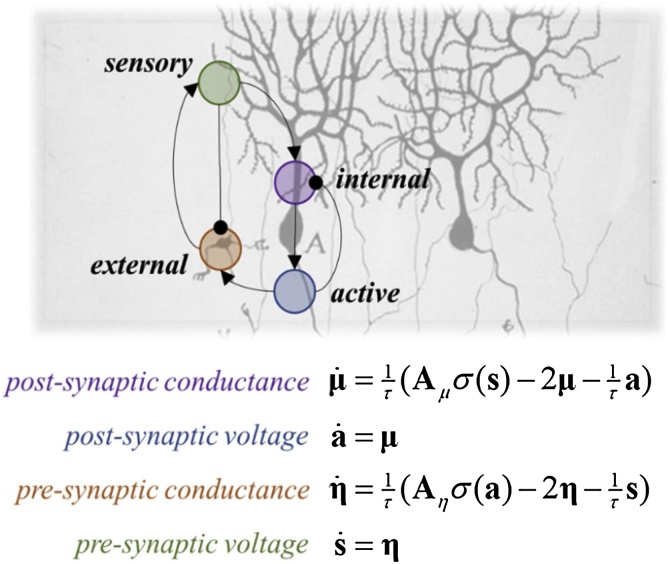
*Neuronal Markov blankets*. This figure illustrates a Markov blanket separating the membrane conductances of a pair of neurons (or between one postsynaptic neuron and all presynaptic neurons). The **A** terms here are constants that act as connectivity strengths from the active state of one neuron to the external state of another (**A***_η_*), and from the sensory states of the latter to the internal states of the former (**A***_μ_*). When many neurons are in play, this becomes a connectivity matrix. The σ-function is a sigmoid shape and can be thought of as converting potentials to firing rates. An interesting feature of this structure is that the sensory states, from the perspective of a given neuron, can arise from many different external states (other neurons) while the active states (membrane depolarisation) depend only on the conductance (internal state) of the neuron being depolarised. Normal arrowheads indicate an excitatory influence, while round arrowheads show inhibitory influences.

Fig. 3 shows explicitly how synaptic dynamics conform to a Markov blanket. This is based upon the neural dynamics used in dynamic causal modelling of canonical microcircuits (Bastos, Usrey et al. 2012; Moran, Pinotsis et al. 2013). This is one of many models of neural dynamics. We have summarised common alternatives – with varying degrees of biophysical detail – in Table 1. As noted above, the existence of a Markov blanket implies a partition of states into external, sensory, active and internal states. The dynamics set out in Fig. 3 assign these labels to the variables that conform to Eq. (2) – i.e., internal states evolve based upon internal and blanket states but not external states, active states do not depend upon external states, and so on.

**Table 1:** Neural models and their blankets.

Model	Dynamics	States	Citation
Hodgkin–Huxley	$\begin{array}{c} \dot{a}=\frac{1}{C}\left(s-\bar{g}\mu^{n}\cdot (a-v)\right)\\ \dot{\mu }=\alpha (a)(1-\mu )-\beta (a)\mu \\ \dot{s}=f_{s}(\eta )\\ \dot{\eta }=f_{\eta }(a,s,\eta ) \end{array}$	**a** – Membrane potential	(Hodgkin and Huxley, 1952)
		**μ** – Ion channels	
		**s** – Injected current	
		**η** – External states	
FitzHugh–Nagumo	$\begin{array}{c} \dot{a}=a-\frac{1}{3}a^{3}-\mu +s\\ \dot{\mu }=\frac{1}{\tau }\left(a+\alpha -\beta \mu \right)\\ \dot{s}=f_{s}(\eta )\\ \dot{\eta }=f_{\eta }(a,s,\eta ) \end{array}$	**a** – Membrane potential	(FitzHugh, 1955; Nagumo, Arimoto et al. 1962)
		**μ** – ‘Recovery’ variable	
		**s** – Injected current	
		**η** – External states	
Morris–Lecar	$\begin{array}{c} \dot{a}=\frac{1}{C}\left(s-\bar{g}\mu \cdot (a-v)\right)\\ \dot{\mu }=\frac{1}{2\tau (a)}\left(1+\tanh\left(\frac{1}{u}\left(a-v\right)\right)-2\mu \right)\\ \dot{s}=f_{s}(\eta )\\ \dot{\eta }=f_{\eta }(a,s,\eta ) \end{array}$	**a** – Membrane potential	(Morris and Lecar, 1981)
		**μ** – Potassium channels	
		**s** – Injected current	
		**η** – External states	

It is worth noting that Markov blankets do not trivially correspond to the boundaries of neuronal cells. Rather, the idea is that a Markov blanket ensures the influences of blanket variables (here, membrane potentials) vicariously enable internal and external states (ion channel conductance) to communicate. This is fundamental because it means that internal and external states, though not influencing each other directly, are the common units that, when coupled, will determine large-scale network behaviour. Moreover, as the blanket is defined in terms of dynamics as opposed to physical boundaries, which would correspond to the cell membrane at the neuronal level, we start to see how the same formalism applies even in the absence of clear spatial boundaries (Friston, 2013; Kirchhoff et al., 2018). At the neuronal level of description, the Markovian demarcation is not insulation of internal states, but rather a way of highlighting (statistically) which states are relevant for self-organisation (Friston, 2019a, 2019b; Hipólito, 2019). Ultimately, the dependencies induced by Markov blankets create a circular causality^5^ : external states, such as the presynaptic conductance, cause changes in internal states, such as the postsynaptic conductance, via sensory states, i.e., presynaptic voltage, while the internal states couple back to the external states through active states, i.e., the postsynaptic voltage.

## 3 Blanket asymmetries

This section deals with the way in which neurons – the basic units of Section 2 – can be connected together to form microcircuits (David and Friston, 2003; Moran et al., 2013; Friston et al., 2019; Coombes and Byrne, 2019), which form the basic unit of Section 4. Specifically, we emphasise the key role of asymmetric interactions between blanketed structures. First, we take a step back to briefly highlight the way in which neurons are studied in isolation. Neurons – as complex, dynamic systems – are highly sensitive to initial conditions, exhibiting organised patterns that result from localised interactions without centralised control. These non-linear interactions can be studied through electrophysiological experiments on single neurons. Typically, this means using voltage clamp experiments and injecting electrical currents. A few examples of physiologically detailed models – to account for these non-linear interactions – are outlined in Table 1 and include the Hodgkin-Huxley model. This has many moving parts and is therefore rarely used in studies of connected neural populations – where dynamics more akin to those in Fig. 3 predominate – but is a good starting point in understanding how sensory states influence the internal state dynamics. This will be essential when we move to sensory states generated by other neural populations in a network.

Intuitively, the Hodgkin–Huxley model expresses the evolution of the membrane potential under time-dependent input currents in terms of the equivalent electric circuit^6^, with a potential that evolves based upon membrane capacitance and currents. More specifically, the Hodgkin–Huxley equations describe how action potentials in neurons are initiated and propagated through a set of non-linear differential equations that approximates the electrical characteristics of excitable neurons in a continuous-time dynamical system (Douglas and Martin, 1991). Formulating the Hodgkin-Huxley (and other models) in terms of the constituents of the Markov blankets inherent in voltage-clamp experiments allows us to highlight the specifics of the influence of the external states (e.g., electrophysiological setup) via sensory states (injected current) on internal states (ion channels), themselves influencing active states (membrane potential). Unpacking the Equation in Table 1 in terms of the specific ion channels, this is:

(3) $$\begin{array}{l} \dot{a}=\frac{1}{C}\left(s-g_{K}\mu_{K}^{4}(a-v_{K})-g_{Na}\mu_{Na}^{3}(a-v_{Na})-g_{l}\mu_{l}(a-v_{l})\right)\\ {\dot{\mu }}_{i}=\alpha (a)(1-\mu_{i})-\beta (a)\mu_{i},\;i=(Na,K,l)\\ \dot{s}=f_{s}(\eta )\\ \dot{\eta }=f_{\eta }(a,s,\eta ) \end{array}$$

Here, the capacitance (*C*) mediates the influence of an injected current (**s**) and ion channel currents on the membrane potential (**a**). This depends upon the ion channels of the system, i.e., the conductance of the sodium (*Na*), potassium (*K*), and leakage (*l*) channels. These depend upon constants (*g*) and the associated internal states (**μ**). In addition, it depends on the ‘reversal’ potentials for each channel (*v*) which specify the potentials at which the direction of ionic flow reverses. The internal states for each channel evolve based upon the (functions – *α* and *β* – of the) potential, as voltage-gated channels open and close to increase or decrease the magnitude of this flow.

The nonlinearity inherent in Eq. (3) facilitates many interesting biophysical phenomena, including bifurcations and limit cycles (Wang, Chen et al. 2007). However, the purpose of this section is to move towards the dynamics exhibited by populations of connected neurons. This rests on the blanket states that mediate these connections. The first step is to notice that the sensory state for the single neuron described by the Hodgkin-Huxley model is an experimental intervention (e.g., an electrophysiologist, **η**) who injects current and measures the resulting potential. We need to move to a situation where input this comes from other neurons. This is afforded by the equations of motion in Fig. 2 for a pair of neurons.

To understand the way in which blankets connect to one another, it is useful to consider that the membrane potential (active state) of a given neuron can only be directly influenced by the conductance (internal states) of that neuron. In contrast, the presynaptic potentials (sensory states) of many other neurons contribute to the internal states. This asymmetry in the blanket states recapitulates that seen in physical systems. Specifically, the position of many different particles (sensory states) can influence the momentum (internal state) of a single particle. However, the position of the particle in question (active state) is only influenced by the momentum of that same particle. This suggests a clear analogy between Newtonian mechanics and neuronal mechanics. Newton’s second law denotes that the rate of change of momentum of a body is directly proportional to the force applied. Conversely, this change in momentum takes place in the direction of the applied force, which itself can depend on position (e.g., the force due to a spring). Rewriting this law, from the perspective of a single particle, in terms of a Markov blanket partition (Friston, 2019a, 2019b), we have:

(4) $$\begin{array}{l} \dot{a}=\frac{1}{m}\mu \\ \dot{\mu }=F(s,a) \end{array}$$

For a single particle, **a** and **μ** are each 3-dimensional (each spatial dimension), while **s** can be many-dimensional, as each particle it describes will have three degrees of freedom. The second law of motion is consistent with neural mechanics in terms of dynamical functions described here in the sense that they both exhibit asymmetrical flow dependencies. This ubiquitous asymmetry is the key to moving to larger spatial scales, and networks of neurons in section 4. This rests upon the structure in Fig. 5, which shows the asymmetric connectivity structure between cortical columns. The neurons, which each include conductance and potential variables, now themselves become parts of sensory, active, internal, or external states with respect to a cortical column. The asymmetry now manifests in forward and backward connections along cortical hierarchies.

## 4 Cortical columns and networks

This section deals with how the same Markov blanketed structure is recapitulated at a larger spatial scale: the cortical microcircuit. Neurons are themselves components of complex self-organising systems. A key characteristic of such complex systems is that they are greater than the sum of their parts: i.e., the properties of a complex system cannot be sufficiently understood from the level of individual components. In the present context, the brain cannot be sufficiently understood from the perspective of interactions between individual neurons. Here, we appeal to the canonical microcircuit model that, not only uses the dynamics of Fig. 3, but connects the neural populations as schematized in Fig. 5. In brief, this divides neural populations into superficial and deep pyramidal cells (which turn out to be blanket states), spiny stellate cells and inhibitory interneurons.

In this section, we use the cortical microcircuit as an example system. This is motivated partly by the ubiquity of this stereotyped network in empirical modelling studies. In turn, this focus on the cortex in empirical work is likely due to the ease with which non-invasive imaging modalities (e.g., electroencephalography and magnetoencephalography) can measure cortical activity—being the closest to the surface of the scalp. However, our aim is not to further a ‘cortico-centrist myopia’ (Parvizi, 2009). The same organisational patterns could just as easily have been identified in subcortical networks—using the homologous equations of motion applied in models of the basal ganglia and thalamus (van Wijk et al., 2018).

Focusing on the canonical microcircuit model has several advantages. First among these is the fact that it is used practically in the analysis of empirical brain data. This is because it can be used to specify models of (i.e., hypotheses about) distributed responses – as measured with functional magnetic resonance imaging (fMRI) or electroencephalography (EEG) – that are physiologically grounded (Friston et al., 2019). For example, it is possible to specify architectures in terms of their forward and backward connections and experimental effects either as extrinsic (between region) or intrinsic (within-region) connectivity at a specific level. A third advantage is that these models enable the assimilation of data from different imaging modalities in the form of multimodal Bayesian fusion (Wei et al., 2020).^7^

Many questions about functional integration the brain benefit from the segregation into the functional units (cortical columns) offered by these microcircuits. A good example is the case of schizophrenia, in terms of the dysconnection hypothesis (Yang et al., 2015; Friston et al., 2016; Kehrer et al., 2008). The dysconnection hypothesis pertains to the functional disintegration of different brain regions, usually based on NMDA-hypofunction models of pathophysiology. This disintegration has dramatic effects on both cortical neuronal and network activity. This hypothesis cannot be framed without knowing what is being disconnected from what. Similarly, questions about cognitive (e.g., attentional) function in health depend upon the same construct (Limanowski and Friston, 2019). Specifically, attention is often conceptualised in terms of selective gain on the influence from one brain region to another. This conceptualisation is only meaningful when we are able to disambiguate pairs of regions from one another (and the rest of the brain). Other important questions, framed using the canonical microcircuit, include questions about the nature of neurovascular coupling. For example, does it depend upon afferent presynaptic activity from extrinsic sources or (only) report to local activity mediated by recurrent (intrinsic) connectivity (Jafarian, Litvak et al. 2020)?

The brain organises itself in a decentralised way. A decentralised system, under complex systems and dynamic modelling theory, is a system whose lower-level components operate on local information to accomplish goals, i.e., control is distributed. The decentralised control is distributed such that each component of the system is equally responsible for contributing to the global, complex activity based on the component's interaction with other components (Deco et al., 2008; Chialvo, 2010; Zuo et al., 2010; Gliozzi and Plunkett, 2019; Hipólito and Kirchhoff, 2019).

Markov blankets allow us to delineate the microcircuitry connections by nuancing their intrinsic connections and how they may also change within the same network. Laminar specific connections underlie the notion of canonical microcircuit (Bastos et al., 2012). As seen in Fig. 5 (second row), we can use the dependencies of this connectivity structure to provide a principled segregation into regions. Considering two columns – connected to one another – we see that if the internal and external states comprise the spiny stellate cells and interneurons of each column, the superficial pyramidal cells of one column act as the active states, while the deep pyramidal cells of the second become sensory states. Unpacking this in detail, the absence of spiny stellate or interneuron connections to the superficial pyramidal cells of other columns is consistent with the absence of influence of external on active states. The reciprocal influence is in place – allowing active states to change external states. Similarly, connections from deep pyramidal to interneurons and superficial pyramidal cells in other columns matches the directed influence of sensory over internal and reciprocated influence between sensory and active states, respectively.

**Fig. 4:**
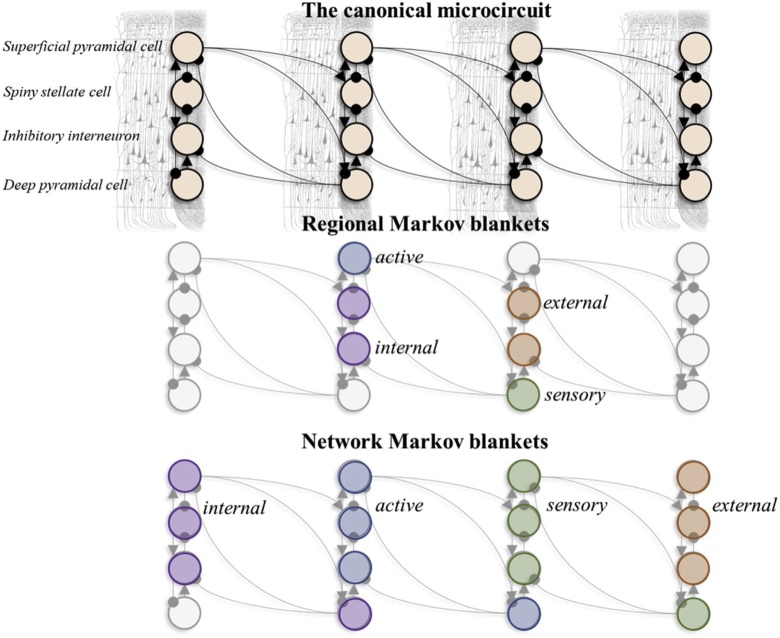
*Cortical micro-circuitry*. The upper schematic shows the connectivity of the canonical microcircuit as employed for DCM (Bastos et al., 2012). This comprises four cell populations with a stereotyped pattern of connectivity. From left to right, we show forward (ascending) connections. The opposite direction shows descending connections. The dynamics of each neural population shown here obey the equations given in Fig. 3, where the likelihood mappings (or **A-**matrices) in those equations specify which populations are connected to one another. As further shown by Bastos et al. (2012), feedforward connections originate predominantly from superficial layers and feedback connections from deep layers, thus suggesting that feedforward connections use relatively high frequencies, compared to feedback connections. The second row here shows the Markov blankets that underwrite the separation into distinct cortical regions (where the superficial and deep pyramidal cells play the role of active and sensory states respectively), and the final row shows a separation into a network of regions, where the middle two regions act to insulate the far left and right regions.

What the Markov blankets in Fig. 4 show is that, while a certain sparsity mediates interactions via blanket states, the internal states of a canonical microcircuit show strikingly interconnected intrinsic architectures. In other words, we can highlight – via Markov blankets – the interconnections between the neurons of origin and termination by highlighting intrinsic connectivity and extrinsic projections. This allows us to determine how top-down and bottom-up processing streams are integrated within each cortical column. As we see in Fig. 4, the top-down stream can be cast as sensory states, and the bottom-up, as active states, both components of the blanket. However, the important aspect of this is the implicit asymmetry. By reversing the ‘internal’ and ‘external’ labels, we could take an alternative perspective and see active as descending and sensory as ascending. Ultimately, this emphasises that intrinsic (local) behaviour is highly dependent upon extrinsic (global) behaviour via specific pyramidal populations. In short, organised patterns are observed as resulting from localised interactions without centralised control. This observation is recapitulated when we zoom out further.

**Fig. 5:**
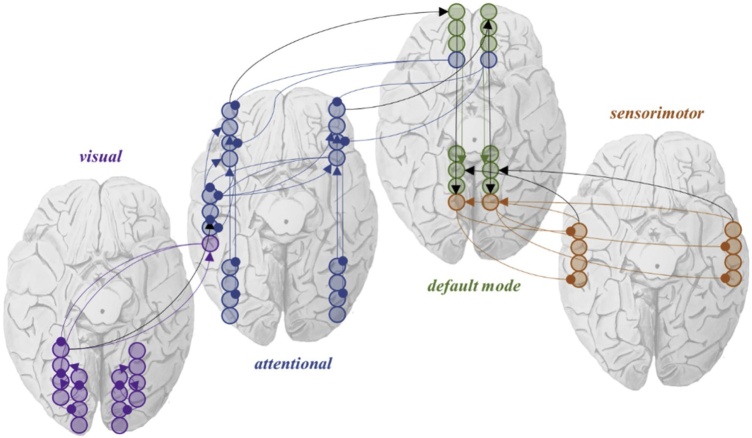
*A Markov blanket of networks*. The image in this figure takes the ideas from Fig. 5 one step further and shows how we could treat the connections between nodes in different networks as dependencies between states in a Markov blanketed system. Here, the networks themselves become the active, sensory, internal, and external states. This graphic is loosely structured around the kinds of networks identified using resting-state fMRI (Razi et al., 2015; Sharaev et al., 2016; Betzel et al., 2014). However, the specific connections and anatomy shown here should not be taken too seriously. Here we treat the visual networks as internal states that reciprocally influence active states (dorsal and ventral attention networks). The default mode network then plays the role of the sensory states, which mediate the influence between the above and external (sensorimotor network) states. The assignment of these is equally valid if reversed, such that sensorimotor networks become internal and visual external.

Zooming out to a larger spatial scale, neuronal structures can be viewed as higher-order neural packets (Yufik and Friston, 2016); i.e., as functional, larger-scale assemblies of neural packets, wrapped in their own superordinate Markov blankets. This is illustrated in the final row of Fig. 4, where cortical columns now become the functional units comprising the states of a Markovian partition to define a network. Fig. 5 takes this one step further, and expresses brain-wide networks as active, sensory, internal, and external states. Bounded assemblies at larger spatial scales are formed spontaneously, consistent with the self-organisation of complex systems defined as structures that maintain their integrity under changing conditions. Especially in approaches such as the one we suggest here, where coordination, segregation and integration are crucial for the self-organisation of the brain as a complex dynamic system.

Taking things a step further, Fig. 5 expresses brain-wide networks as active, sensory, internal, and external states. This is to emphasise that there may be entities comprising multiple brain networks whose interactions with one another conform to the same conditional dependency structure as the regions within those networks (and the microcircuits within those regions). Markov blankets of networks may be the intermediate step between those within networks, and the blankets mediating interaction between different brains (Bilek, Zeidman et al. 2020).

Blankets that bound microcircuits are then providing the units that make up larger scale assemblies. This can be seen as blankets of blankets or as a nesting of blankets. A treatment of the emergence of intrinsic brain networks and critical dynamics (nested blankets) has been offered in Friston et al. (2020a, 2020b) by using the *renormalisation group*, it is shown that much of the phenomenology found in network neuroscience is an emergent property of a particular partition of neuronal states, over progressively coarser scales, such as larger scale assemblies. Markov blankets allow us to articulate neuronal assemblies, as flexible but also stable biophysical structures. In other words, structures such as these maintain their integrity under changing conditions. In this treatment, Markov blankets highlight the assemblies conserved over multiple levels of description, i.e., they are scale-free. Monitoring the variations in such larger spatial scales enables attributing to neurons, microcircuits, and networks the ability to undergo changes without loss of self-identity.

## 5 Discussion

The crucial point for any system, at any scale, is that its boundaries are dictated by conditional dependencies that depend upon certain states. By role, we mean the way in which systemic states induce changes in other states. For example, active post-synaptic potentials induce changes in *sensory* presynaptic conductances, and *active* superficial pyramidal cells induce changes in *sensory* deep pyramidal cells. These identities determine the form of segregation from, and interaction with, other parts of the brain. Given the centrality of interacting subsystems in neurobiology, it is vital to know what interacts with what at each level of analysis. It is by their flexibility that Markov blankets allow us to explain functional integration while still drawing statistical boundaries. Markov blankets demarcate boundaries of couplings from pairs of neurons, to cortical columns and brain-wide networks. The description of neural connectivity with Markovian formalisms allows zooming in and out, identifying different functional units at different scales. The persistence of Markov blanketed structures over time has a further interesting consequence. Such systems may be shown to behave according to a Bayesian mechanics (Friston, 2019a) in which internal state dynamics may (on average) be expressed as gradient flows on Bayesian model evidence – or a bound on this quantity known as variational free energy.^8^

This has three practical consequences. The first is that it provides a conceptual endorsement of empirical approaches such as dynamic causal modelling (DCM), which depends upon characterisation of effective connectivity between functionally segregated neural circuits. In brief, DCM rests upon two components: biophysical modelling using differential equations and Bayesian statistical methods for model inversion (parameter estimation) and comparison. DCM has many practical applications in analysing brain data acquired under a range of paradigms. For example, it has been used in the study of attentional modulation during visual motion processing (Büchel and Friston, 1997; Friston and Price, 2003), in multisensory integration (Limanowski and Friston, 2019), and in studies of clinical conditions (Dietz, Friston et al. 2014). Its role is to disambiguate between different hypotheses about how experimental conditions (like attentional set) modulate neuronal connectivity. With non-linear dynamic causal models (Stephan et al., 2008), non-linear DCM for fMRI enables the modelling of how activity in one population gains connection strengths, among others.

The Markovian formalism provides a flexible calculus to accommodate co-existing and interacting elements, which play important roles for the optimal functioning of the system. It enables us to look at the organism by considering each and every level of complexity, without losing the unity of the simplest component. Neurobiology spans from the small scale of molecular biology to the social and environmental aspects of pathology; how to accommodate these different aspects in an inter-scale manner is the key challenge, and what we are proposing is a promising tool to meet this challenge.

Once Markov blankets have been drawn, the neurons, cortical columns and networks, they all appear to dynamically self-organise under a common principle: the free-energy principle (Friston, 2013). This says that any self-organising system will selectively interact with its environment to minimise free energy, thereby resisting the natural tendency to disorder and entropy. This paper has sought to identify the Markov blankets in the brain. In future work, we hope to unpack these blankets in terms of active inferential processes, where post-synaptic ion channels may be seen as inferring pre-synaptic channels (Kiebel and Friston, 2011), stellate cells and interneurons inferring their counterparts in other cortical columns, and groups of columns in a network inferring the internal states of other networks.

The treatment of neurons as if they were active agents, drawing inferences about their environments, has precedence in existing theoretical work. For example, Kiebel and Friston (2011) demonstrated how dendrites can self-organise to minimise a variational free-energy bound on surprise of their presynaptic inputs, demonstrating that postsynaptic gain is itself optimised with respect to variational free-energy. This provides a principled account of neuronal self-organisation built upon the optimisation of elemental neuronal (dendritic) processing. This agenda has subsequently been developed in theoretical (Palacios et al., 2019) and empirical (Isomura and Friston, 2018) studies of neuronal self-organisation.

Anticipatory mechanisms are shared by all living systems. Indeed, for an organism to remain alive, it must regulate – and therefore anticipate – the structure of its exchanges with its embedding environment, which evinces a role for *prediction*. In some organisms, especially those animals that possess a nervous system, anticipatory mechanisms are evident in patterns of organised behaviour and are made particularly evident by whole-brain dynamics over longer timescales. This motivates a specific research agenda in computational neuroscience: to investigate how microcircuits organise (and why they reorganise) on the local level and smaller, micro scales, crucially, without losing sight of the embodied brain.

It is important to recognise the limitations of this paper. While we have outlined how dynamic Markov blankets may be identified, we have done so with known equations of motion. When these are not known, as in most practical settings, the interactions between variables must be estimated. In addition, we have largely restricted our conceptual analysis to how we partition systems into fundamental (at a given scale) units. The next steps will be to unpack the consequences of this partition both analytically and through numerical simulation, with a view to the variational inferential perspective touched upon in the discussion. We have provided the foundation for this, as once we know the external and blanket states, we know what the internal states must be ‘inferring’. This offers a well-formed scientific question as to the form of the implicit model the internal states use to engage in active inference – i.e., how do external states give rise to sensory states? Part of this work will be to ask questions about how brain networks self-organise. Finally, we hope to apply these ideas to the study of neuropsychiatric conditions. Of special interest would be to develop experimental work on the span from neurobiology to social and environmental aspects of pathology, which is still missing a unifying link.

## Conclusion

This paper introduced the characterisation of neural systems as depending upon a boundary – or Markov blanket. That is a mediation of the interaction between what is inside and outside of a system. This treatment was illustrated using the canonical micro-circuitry used in empirical studies of effective connectivity, to directly connect this analysis to models used in neuropsychiatric and computational psychiatry research (Frank et al., 2016; Shaw et al., 2020). The key point is that brain function depends upon the cooperative dynamics of networks, regions, and neurons. To talk meaningfully about these units of self-organisation, we need a principled means of partitioning neuronal states into one unit or another. This partition is afforded by the dependency and flow structure of a Markov blanket, whose statistical form is recapitulated across each level of analysis. This endorses the partition of neural systems at each of these stages (e.g., into neurons, regions, networks etc.), but also highlights the limitations of 'modular' perspectives on brain function that only consider a single level of description. In short, the level of analysis we choose to adopt defines a Markov blanket that operationally defines the appropriate functional units we need to consider. In all cases, these can be broken down into four classes of variable: active, sensory, internal, or external. In this light, the physics of the mind is consistent with the "enactive" view (Hipólito, 2018), deriving cognition from an interplay between external conditions and self-organisation in the nervous system.

## Glossary of Terms

Canonical microcircuit: Distributed network of relatively simple elements that give rise to complexity of cognitive processing by virtue of (1) their extensive interaction with other elements; and (2) their own intrinsic rich circuits. Originally introduced by (Douglas and Martin 1991) as a functional motif of interconnected neuronal populations that is considered to be replicated over the cortical sheet.
Complex system: a system that is composed of many components which may interact with each other. Examples include Earth's global climate, organisms the human brain, or living cells. Their behaviour is particularly difficult to model due to the dependencies and relationships between their parts and the system with the environment.
Decentralised system: local interactions between components of a system establish order and coordination to achieve global goals without a central commanding influence. Interactions are formed and predicated on spatiotemporal patterns, which are created through the positive and negative feedback that interactions provide.
Dynamic causal modelling: modelling treatment of neural dynamics as a non-linear dynamic system. Differential equations describe the interaction of neural populations, which direct or indirectly give rise to functional neuroimaging data, particularly by parameterising directed influences or effective connectivity, usually estimated using Bayesian methods.
Emergence: traits of a system that are not apparent from its components in isolation, but which result from the interactions, dependencies, or relationships they form when placed together in a system. These components are impossible to predict from the smaller entities that make up the system.
Neural mass models: models of coarse-grained activity of large populations of neurons and synapses especially useful in understanding brain rhythms and synchronisation.
Non-linearity: Non-linearity describes systems with high dependence on initial conditions, current state, and parameter values. The differential equations of non-linear dynamical systems are non-linear in the states (and parameters; in other words, they have high order terms beyond linear coupling.
Relative entropy: mutual information, or the uncertainty about particular states minus the uncertainty, given the external states. In other words, the information gained about one set of states, given another.
Self-entropy: entropy of particular states, i.e., of states that constitute a particle, namely autonomous and sensory states. Entropy is a measure of uncertainty, disorder or dispersion.
Self-organisation: a process of spontaneous pattern formation across time scales – from microscopic cells to macroscopic organisms – that entails the emergence of stable systemic configurations that distinguish themselves from their environments.

## Footnotes

1 We will occasionally appeal to technical terms that are in common usage in this field. Please see the glossary of terms for definitions.

2 Markov blankets, under the Free Energy Principle, can be understood as pertaining to self-organizing systems whose dynamics – on average – to minimize a variational free energy functional.

3 We should note that our interpretation of the Markov blanket as a causal construct might be controversial to some. Some authors employ a less restrictive definition of a Markov blanket and take this to be the minimal set of variables that satisfies the conditional independence relationships in Eq. (1) (Hausman and Woodward, 1999), without reference to the constraints on dynamical coupling in Eq. (2).

4 The Markovian formalism has been used also in the context of structural equation modelling. We are interested in dynamical, as opposed to static, systems, however. Give this, dynamic systems approaches, such as dynamic causal modelling and neural mass models, are more appropriate for our purposes.

5 A causal interpretation of the Markov blanket is not uncontroversial. For us to interpret the Markov blanket construct causally in the context of structural equation modelling, the system must conform to the causal Markov condition (Hausman and Woodward, 1999). This would usually be very restrictive (Bongers and Mooij, 2018). However, this is arguably not an issue for the current formulation, which grounds its causal interpretation not in structural equation modelling but in dynamical systems theory, i.e., in the dynamic causal modelling approach, the causal status of which is uncontroversial (Moran et al., 2013).

6 Note that this is not what is meant by the term ‘microcircuit’, which refers to the ‘wiring’ of a population of neurons into a local network.

7 The idea here is that a model of the underlying neurophysiology can be used to make predictions about the measurements that could be obtained using different instruments. As such, if we were to measure the brain using fMRI and EEG, both should inform the same (parameters of the same) model.

8 The free energy principle (FEP) states that self-organizing systems that have a Markov blanket will engage in behaviour that appears to minimize a variational free energy functional.
